# A systematic review and Bayesian meta-analysis assessing intelectin-1 in cancer patients and healthy individuals

**DOI:** 10.3389/fonc.2024.1198555

**Published:** 2024-02-28

**Authors:** D. Robert Paval, Thomas G. Di Virgilio, Richard J. E. Skipworth, Iain J. Gallagher

**Affiliations:** ^1^ Faculty of Health Sciences & Sport, University of Stirling, Stirling, United Kingdom; ^2^ Clinical Surgery, Edinburgh Medical School and Royal Infirmary of Edinburgh, Edinburgh, United Kingdom; ^3^ Center for Biomedicine and Global Health, Edinburgh Napier University, Edinburgh, United Kingdom

**Keywords:** intelectin, omentin, cancer, gastrointestinal, review, Bayesian

## Abstract

**Background:**

Intelectin-1 (ITLN1) is an adipokine with multiple physiological functions, including a role in tumour formation and development. Previous research reported variable ITLN1 levels for cancer patients and healthy individuals. This study aimed to compare ITLN1 concentrations between controls and cancer patients and to determine the adipokine’s physiological level.

**Methods:**

Five databases were searched in January 2022 for studies that measured the level of ITLN1 in adults that were healthy or diagnosed with any type of cancer. After title, abstract and full-text screening, the methodological quality of the studies was assessed. The extracted data were meta-analysed using the R language and Bayesian statistical techniques.

**Results:**

Overall, 15 studies compared circulating ITLN1 levels between healthy individuals (n=3424) and cancer patients (n=1538), but no differences were observed between these studies. ITLN1 was not different between groups in an analysis that evaluated high-quality studies only (n=5). The meta-analysis indicated considerably higher ITLN1 levels in gastrointestinal (i.e., colorectal, pancreatic, gastric) cancer compared to controls, while the other cancer types did not demonstrate differences between groups. The mean ITLN1 level of healthy individuals was 234 ± 21ng/ml (n=136), while the average value in high-quality studies (n=52) was 257 ± 31ng/ml.

**Conclusion:**

Different types of cancer showed different circulating ITLN1 patterns. Circulating ITLN1 concentration was higher in gastrointestinal cancer compared to controls, with strong support from the meta-analytical model. Our analysis also determined the mean ITLN1 level in healthy individuals; this is a crucial starting point for understanding how this cytokine associates with diseases. Two-thirds of the studies were of low methodological quality and thus, future work in this field must focus on improved methods.

**Systematic review registration:**

https://www.crd.york.ac.uk/prospero/display_record.php?RecordID=303406, identifier CRD42022303406.

## 1 Introduction

Intelectin (ITLN) is an immune lectin that contains a fibrinogen-like domain and a unique intelectin-specific region (1, 2). ITLN1 and ITLN2 are two homologs that share 83% amino acid identity. Both ITLN1 and ITLN2 can bind to microbial glycan chains but not to human glycans and thus, ITLN may have a role in antimicrobial defence (2, 3). ITLN is largely produced by stromal vascular fraction cells in VAT (4). Furthermore, low levels of ITLN1 were found in SAT, epicardial fat, lungs, renal collecting tubes, colon and the small intestine, while ITLN2 was primarily expressed in intestinal Paneth cells (2, 5–7).

Previous research indicated that circulating ITLN1 concentrations differ between cancer patients and healthy individuals (8–11). Indeed, in our recent narrative review (12), we observed a difference in circulating ITLN1 levels when people with cancer were compared to healthy individuals. The difference was influenced by cancer type since patients with gastrointestinal and prostate cancer showed higher concentrations of circulating ITLN1, while individuals with breast, bladder, renal and gynaecological cancer expressed lower circulating ITLN1 levels. We also noted a relationship between cancer cachexia and local but not circulating ITLN1 (13). The same study indicated that ITLN1 mRNA and protein concentrations were substantially elevated in the VAT of patients with gastrointestinal cancer. Other research that measured gene expression suggested that tumour levels of ITLN1 were significantly different from the concentrations observed in healthy tissue (10, 14). ITLN1 can activate the PI3K/Akt pathway (15, 16) and the improper regulation of this pathway can determine a cascade of events that favour cancer development and progression. Also, obesity was associated with an increased risk of tumour formation (17) and proposed as a factor that influences the relationship between ITLN1 and cancer (18, 19). The decrease of ITLN1 levels observed in overweight and obese individuals could be a marker of the metabolic effects of obesity, contributing to a deregulation of the PI3K/Akt pathway (12). Therefore, ITLN1 could be a potentially important target in cancer biology. Its mechanisms of action as well as concentration differences between cancer patients and healthy individuals should be further explored and explained.

Importantly, the physiological level of ITLN1 remains uncertain as the adipokine’s reported concentrations are highly variable in both cancer patients and healthy individuals. Some variability is to be expected when ITLN1 is measured in cancer patients. Indeed, Arjmand et al. (20) examined the relationship between ITLN1 and cancer in a systematic review and observed that concentrations ranged from 2 to 1100 ng/ml. Whilst comprehensive, their review had some methodological weaknesses that we addressed in a previous study (12). In healthy people, Watanabe et al. (7) reported that circulating ITLN1 levels vary from 5 to 800 ng/ml. This range is close to that reported by Arjmand et al. (20) in people with cancer. There has been no discussion so far about this wide range of ITLN1 values observed across different studies. Determining the source of variability and the physiological concentration of ITLN1 would allow useful comparisons between healthy individuals and patients with different conditions, in which adipokine levels are typically dysregulated. Quantification of a mean physiological concentration would also set the benchmark for future cell culture experiments and animal studies. Therefore, this systematic review aims to compare ITLN1 concentrations between cancer patients and healthy controls and to determine the mean level of ITLN1 in healthy individuals.

## 2 Methods

The protocol of this systematic review is described below and was also registered on PROSPERO on 18/01/2022 (registration number CRD42022303406). Ethical approval was not required for this study.

### 2.1 Search strategy

The following databases were searched for studies published in English on 18/01/2022: MEDLINE, EMBASE, CENTRAL, CINAHL and Web of Science. The search strategy (i.e. intelectin* OR omentin*) was designed to capture all studies measuring intelectin or omentin (including related terms), without imposing any additional criteria. The **Supplementary Material** includes a detailed version of the search strategy and the number of studies extracted from each database.

### 2.2 Inclusion and exclusion criteria

Eligible studies measured circulating and tissue ITLN1 protein levels of adults that were either healthy or diagnosed with any type of cancer. All types of cancer were included in the current review. Studies that examined healthy participants were included if enough details were given to make an objective assessment of participants’ health status. Healthy participants did not have any medical conditions that could affect ITLN1 levels. Studies were not included in the analysis if participants’ health status could not be accurately assessed or if the details given were limited or unclear. No criteria were imposed on the design of the studies as long as a measurement of ITLN1 was included. This review did not include any studies that measured ITLN1 protein in pregnant women or children. Moreover, animal models, protocols or conference abstracts were excluded from the current review.

### 2.3 Study selection and data extraction

**Figure 1:**
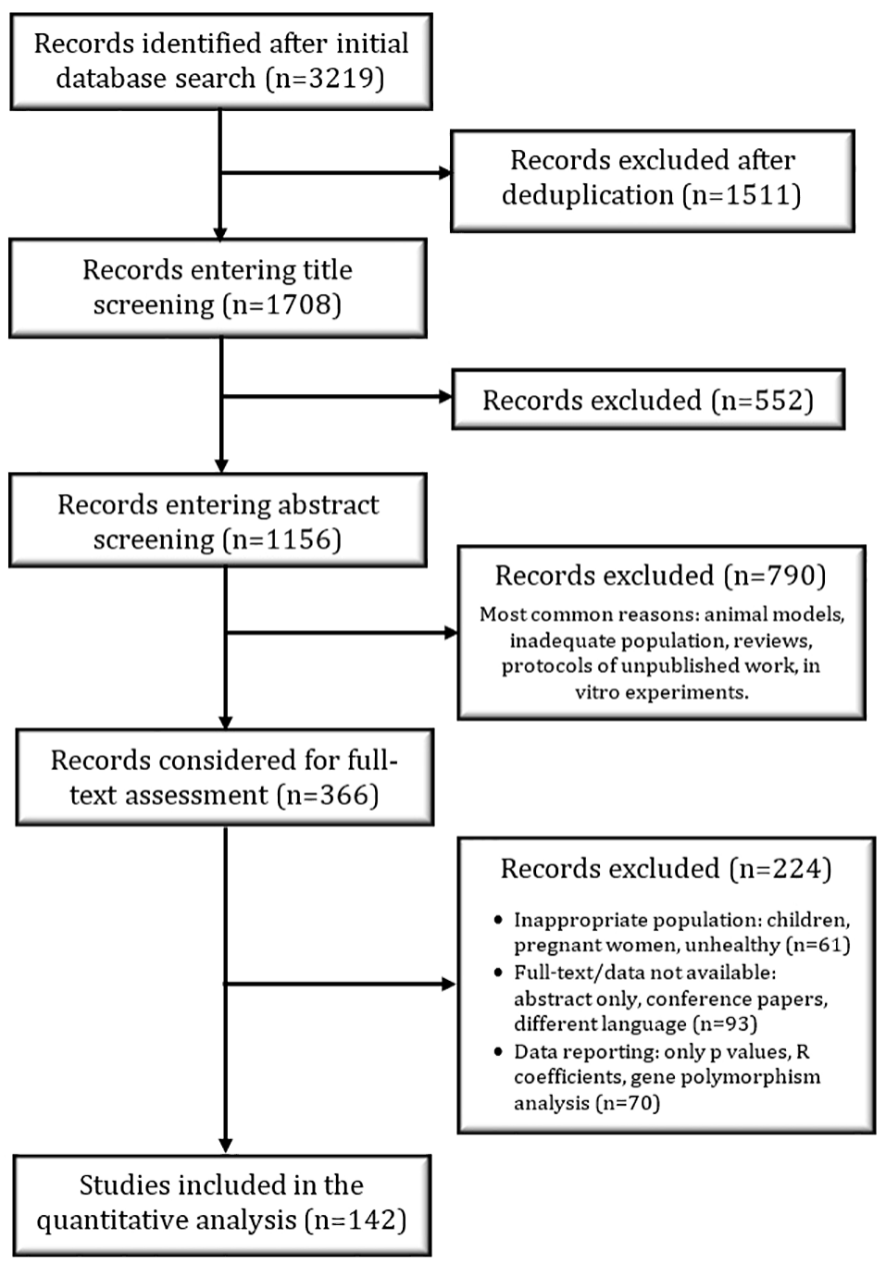
PRISMA flow diagram of the study selection process.

The PRISMA flow diagram (**Figure 1**) highlights the study selection process that was independently conducted by RP. The titles were screened in a conservative manner – if the title did not provide enough information, the study was included in the next phase of selection. A similar approach was used for abstract screening; after this step, all suitable studies were considered for full-text screening. At the end of the full-text screening, relevant data were extracted from the included studies with a specifically designed collection form. The number of participants, the mean age and BMI, the method used to quantify ITLN1, the description of the assay used to quantify ITLN1 and recorded ITLN1 levels were extracted from all studies. Additionally, the type of cancer, the stage of the disease as well as the treatment received by participants were extracted from the studies that evaluated people with cancer. The authors of eligible primary studies were contacted whenever additional data were required.

### 2.4 Study quality and data analysis

Only a small number of studies measured tissue concentration of ITLN1 and thus, the current meta-analysis focused on circulating levels. The timing of the blood sample and the characteristics of the assay used to quantify ITLN1 were extracted and used to assess study quality. Firstly, for the purpose of this meta-analysis, an essential criterion was that studies collected blood in the morning after an overnight fast, as previous research (21, 22) indicated that (adipo)cytokines are affected by diurnal variation. Secondly, given the variable concentration of ITLN1, it was crucial that studies mentioned the assay used to quantify ITLN1, described its characteristics (e.g., sensitivity, range) and reported data that were in accordance with the assay parameters (e.g., within detection limits). The data extracted from studies that adhered to the previously mentioned methodological standards were considered high-quality data. Conversely, studies that failed to report all or any of the characteristics described earlier were not included in the high-quality studies group.

All data were analysed using the R programming language, version 4.2.1 (23). Most of the studies reported ITLN1 in the ng/ml range and used the mean and standard deviation (SD) to report the concentrations. When this was not the case, data were converted to mean and SD (24) and to ng/ml. Throughout this study, data are reported as mean ± SD unless otherwise specified.

The present study used a Bayesian approach to analyse the available data. The R package RoBMA (Robust Bayesian Meta-Analysis) was used to compare standardised mean differences (SMDs) between cancer patients and healthy individuals (25). The package accounts for potential publication bias in the statistical model and uses Bayesian model averaging to compare meta-analytic models that assume the presence or the absence of an effect, heterogeneity and publication bias. Bayes’ factors (BFs) and model-averaged values determined from posterior model probabilities are used to indicate the magnitude of the relationships (26). BFs compare two models and express the relative strength of the evidence in favour of one of the two models that are compared (e.g., null model versus alternative model, experimental group versus control group). Kass and Raftery (27) provide a scale for interpretation of BFs. In this review, the BF indicates the relative evidence that e.g., ITLN1 levels are higher in one group compared to the other. Bayesian model averaging provides a principled method to make probability weighted averages from several possible models (28). In this study the meta-analytic estimate of ITLN1 level was made by averaging over each of the models considered by RoBMA. Furthermore, the package brms (29) was used to determine mean ITLN1 levels in healthy individuals by fitting a normal-normal hierarchical model to the available data (30). Bayesian highest density intervals (HDI) were used to describe and summarise the uncertainty associated with the model-estimated parameters. For example, a 95% HDI represents the range of the posterior distribution that contains 95% of the values determined by the meta-analytical model. The complete dataset and the R code used to examine the data are included at the end of the study in the *Data Availability Statement*.

## 3 Results

### 3.1 Study characteristics

A total of 1708 studies that measured ITLN1 levels were identified after the removal of duplicates and were included in the title screening phase (**Figure 1**). Subsequently, 1156 abstracts were examined, of which 366 records were considered for the full-text assessment. At the end of the screening phases, data from 142 studies were extracted and statistically examined. Of these, 15 studies were included in a meta-analysis comparing ITLN1 levels between healthy individuals and cancer patients. A further 5 studies measured ITLN1 levels in cancer patients but did not include a healthy control group – these were not examined in the meta-analysis but were used to characterise the level of ITLN1 in different types of cancer. Additionally, data from 138 studies that measured circulating ITLN1 in apparently healthy individuals were used to estimate a Bayesian 95% HDI for the physiological level of circulating ITLN1 in healthy individuals.

The characteristics of the studies that measured ITLN1 in cancer patients are described in **Table 1**. Overall, 1538 patients and 3424 controls were included in the meta-analysis that compared ITLN1 levels between cancer patients and apparently healthy individuals. A further 342 patients were recruited in the 5 additional studies that measured circulating ITLN1 in cancer patients but did not include a control group. The mean age of individuals with cancer was 60 ± 7 (mean ± SD), with female patients representing 50% of the sample. The most common types of cancer were breast, colorectal, prostate and pancreatic cancer, although other types such as lung and ovarian cancer were also assessed. Since some cytokines can show intra-day variation (21, 22) and having consistent methodological approaches is essential for meta-analyses, the timing of blood sampling was a factor that played a key role in assessing the quality of the included studies. A total of 10 studies collected blood in the morning after an overnight fast, 3 studies reported that blood was collected in a fasted state (without mentioning the period of the day), 1 study suggested that blood was collected in the morning (but did not indicate whether participants fasted), 3 studies stated non-specific/opportunistic time of blood collection without giving further details (i.e., pre-operative, at hospital admission) and 3 studies did not report collection methods. Furthermore, the majority (19/20) of the studies used an ELISA to quantify the level of circulating ITLN1, with only one study (43) using a multiplex immunoassay (**Table 1**). Overall, only 8 studies adequately described the characteristics of the assay (**Table 1**), while 5 studies did not report any characteristics (e.g., sensitivity, range) and 7 were inconsistent (e.g., reported data were outside the assay range) or failed to give sufficient details (e.g., the manufacturer’s reported intra- and inter-assay coefficient of variation). Interestingly, only 5 studies passed the methodological quality control and provided high-quality data (32, 35, 36, 40, 41) as the assay characteristics were reported adequately and the blood was collected in the morning, after an overnight fast.

**Table 1:** Characteristics of studies that measured circulating ITLN1 in cancer patients.

Study (year)	Cancer type	Participants (n)	ITLN1 level (ng/ml)	Blood collection method	Assay method	Assay details reported
Alaee et al. (2016) (31)	Breast	Patients (30)	73.1 ± 29.7	N/R	ELISA	Inconsistent
		Controls (30)	108.8 ± 65.4			
		Data shown as mean ± SD				
Aleksandrova et al. (2016) (8)	Colorectal	Patients (251)	459 (379-570)	N/R	ELISA	Yes
		Controls (2295)	396 (328-486)			
		Data shown as median (IQR)				
Karabulut et al. (2016) (9)	Pancreatic	Patients (33)	9.6 (3.6-219.5)	At hospital admission	ELISA	No
		Controls (33)	1.6 (0.8-5.0)			
		Data shown as median (range)				
Shen et al. (2016) (32)	Renal	Patients (41)	3.6 ± 0.8	Fasting	ELISA	Yes
		Controls (42)	9.9 ± 1.4			
		Data shown as mean ± SD				
Zhang et al. (2016) (33)	Bladder	Patients (42)	1.8 (0-9.2)	Overnight fast	ELISA	Inconsistent
		Controls (42)	5.2 (2.3-13.2)			
		Data shown as median (IQR)				
Yildiz et al. (2017) (34)	Ovarian	Patients (41)	43.8 ± 19.1^	N/R	ELISA	Yes
		Controls (41)	37.4 ± 12^			
		Data shown as mean ± SD				
Khademi-Ansari et al. (2018) (35)	Lung	Patients (45)	3.6 ± 0.7*	Fasting	ELISA	Yes
		Controls (31)	3.6 ± 0.6*			
		Data shown as mean ± SD				
Kiczmer et al. (2018) (36)	Pancreatic	Patients (20)	582.5 (422.6-663.7)	Overnight fast	ELISA	Yes
		Controls (18)	461.7 (345.4-494.4)			
		Data shown as median (IQR)				
Nourbakhsh et al. (2018) (37)	Breast	Patients (45)	157 ± 66*	Overnight fast	ELISA	No
		Controls (45)	217 ± 75*			
		Data shown as mean ± SD				
Zhao et al. (2019) (38)	Colorectal	Patients (358)	67.3 ± 32.3	Overnight fast	ELISA	Insufficient
		Controls (286)	33.2 ± 20.0			
		Data shown as mean ± SD				
Zhou et al. (2019) (11)	Prostate	Patients (90)	12.9 ± 6.15	Overnight fast	ELISA	Inconsistent
		Controls (90)	5.0 ± 4.7			
		Data shown as mean ± SEM				
Feng et al. (2020) (39)	Colorectal	Patients (319)	69.3 ± 23.5	Overnight fast	ELISA	No
		Controls (300)	37.9 ± 15.4			
		Data shown as mean ± SD				
Miller et al. (2020) (13)	Gastrointestinal	Patients (12)	3.8 ± 6.4	At induction of anaesthesia	ELISA	Yes
		Patients (12)	1.9 ± 0.8			
		Controls (12)	2.3 ± 1			
		Data shown as mean ± SD				
Christodoulatos et al. (2021) (40)	Breast	Patients (103)	340.5 ± 109.3	Overnight fast	ELISA	Yes
		Controls (103)	476.7 ± 156.1			
		Data shown as mean ± SD				
Panagiotou et al. (2021) (41)	Breast	Patients (72)	567.7 ± 236.2	Overnight fast	ELISA	Yes
		Patients (24)	589.0 ± 256.3			
		Controls (56)	436.6 ± 173.7			
		Data shown as mean ± SD				
No control group						
Uyeturk et al. (2014) (42)	Prostate	Patients (50)	547.8 (297.1-945.7)	Overnight fast	ELISA	Inconsistent
		Data shown as median (range)				
Cymbaluk-Ploska et al. (2018) (43)	Endometrial	Patients (92)	610.1 (218.5-13377)	Pre-operative	Multiplex immunoassay	No
		Data shown as mean (range)				
Fryczkowski et al. (2018) (44)	Prostate	Patients (40)	478.8 (398.2-584.7)	Overnight fast	ELISA	Inconsistent
		Data shown as median (IQR)				
Borowski and Sieminska (2020) (45)	Prostate	Patients (72)	594.3 ± 266.9	Morning	ELISA	No
		Data shown as mean ± SD				
Tahmasebpour et al. (2020) (10)	Breast	Patients (88)	132.3 ± 9.1*	Fasting	ELISA	Insufficient
		Data shown as mean ± SD				

SD, standard deviation; IQR, interquartile range; ELISA, enzyme-linked immunosorbent assay.

*Data reported as ng/l.

^Data reported as pg/ml.

### 3.2 ITLN1 differences between cancer patients and healthy individuals

**Figure 2:**
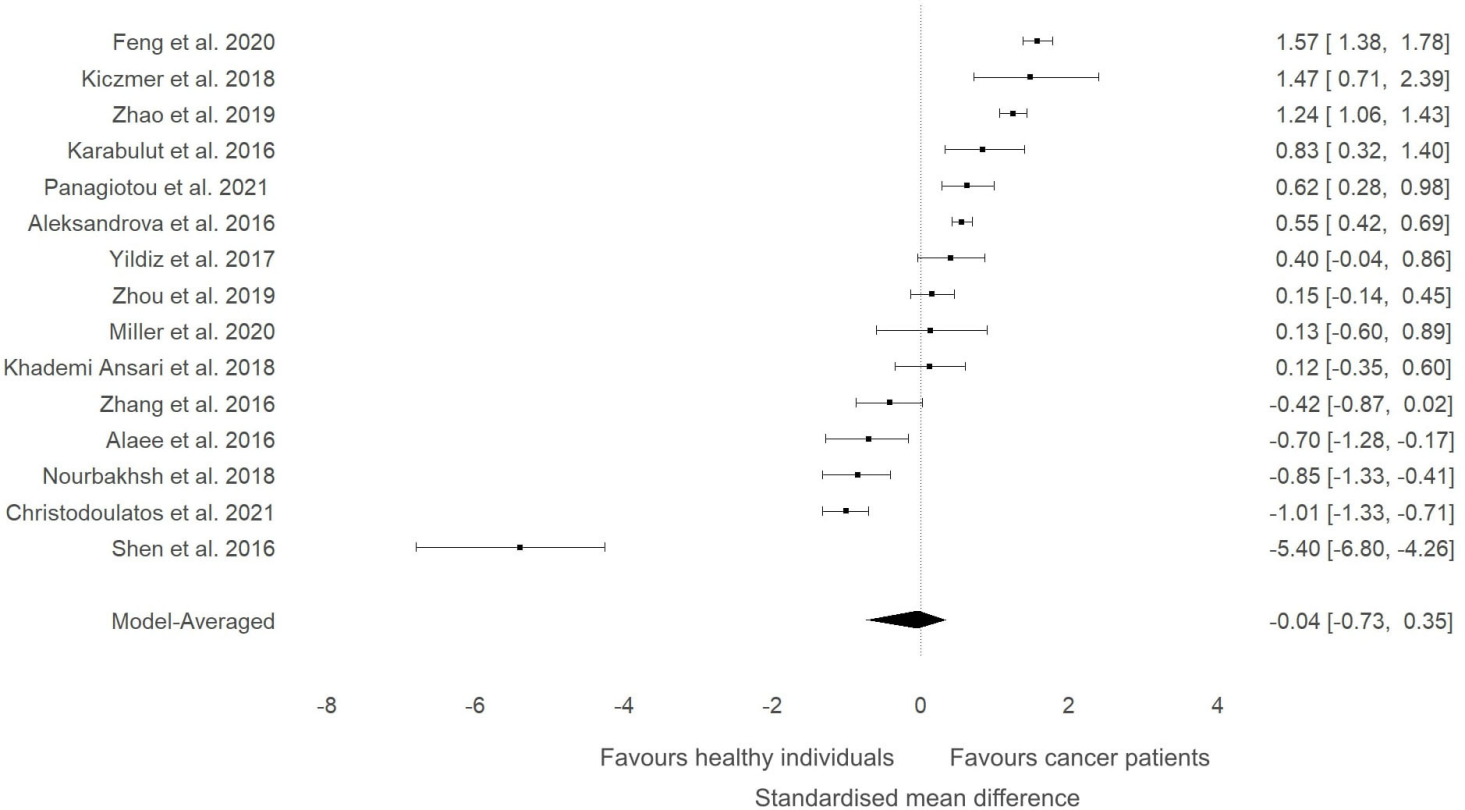
Forest plot showing meta-analytic results for the comparison of ITLN1 levels in cancer patients and healthy individuals. Data are presented as SMD and 95% HDI.

A meta-analysis was conducted to evaluate whether the circulating levels of ITLN1 varied between healthy individuals and cancer patients (**Figure 2**). The analysis found weak evidence (27) in favour of higher ITLN1 values in the control group (BF=0.4) when all types of cancer were examined (SMD=-0.04; 95%HDI: -0.74 to 0.35). Additionally, there was a very high level of evidence in favour of heterogeneity (BF>10000) but low evidence of publication bias (BF=0.5).

**Figure 3:**
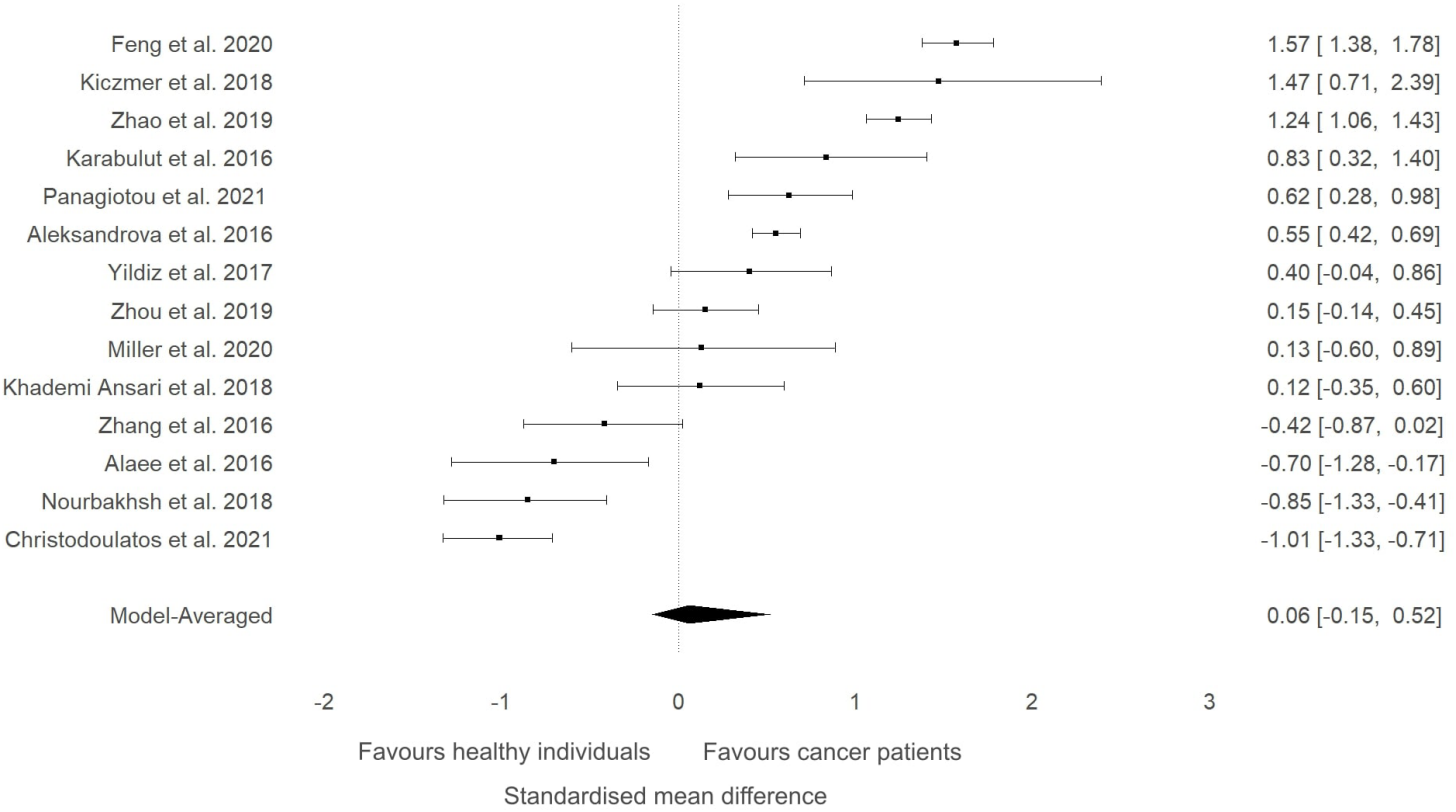
Forest plot showing meta-analytic results of the sensitivity analysis for the studies that compared ITLN1 levels in people with cancer and healthy individuals after excluding the outlying study by Shen and colleagues (46). Model-averaged data are presented as SMD and 95% HDI.

**Figure 4:**
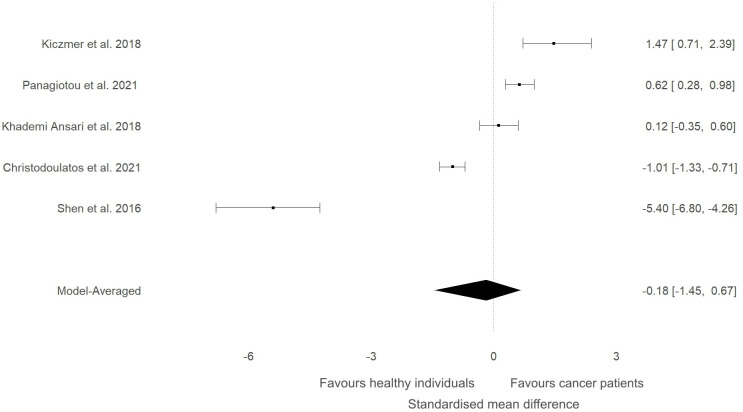
Forest plot showing meta-analytic results for high-quality studies that compared ITLN1 levels in people with cancer and healthy individuals. Data are presented as SMD and 95% HDI.

A sensitivity analysis was conducted to check if an outlying study (32) had an impact on the overall results (**Figure 3**). Although a minor change was observed in the SMD between groups (SMD=0.06, 95%HDI: -0.15 to 0.52) after removing the study, the evidence in favour of a difference between groups was still weak (BF=0.4). The level of heterogeneity was consistently high (BF>10000), while the evidence of publication bias remained weak (BF=0.9). Consequently, the study by Shen and colleagues (32) did not have a considerable impact on the overall results of the current meta-analysis. High-quality studies were extracted and analysed separately to examine if study quality had an impact on the meta-analytic results. The mean effect size (SMD=-0.18, 95%HDI: -1.45 to 0.67) and the strength of the available evidence (BF=0.8) was not substantially different from the original model, indicating that ITLN1 levels are not different when all people with cancer are compared to healthy individuals (**Figure 4**). Similar to the previous models, there was strong evidence in favour of a high degree of heterogeneity (BF>10000) and low support for publication bias (BF=0.7).

**Figure 5:**
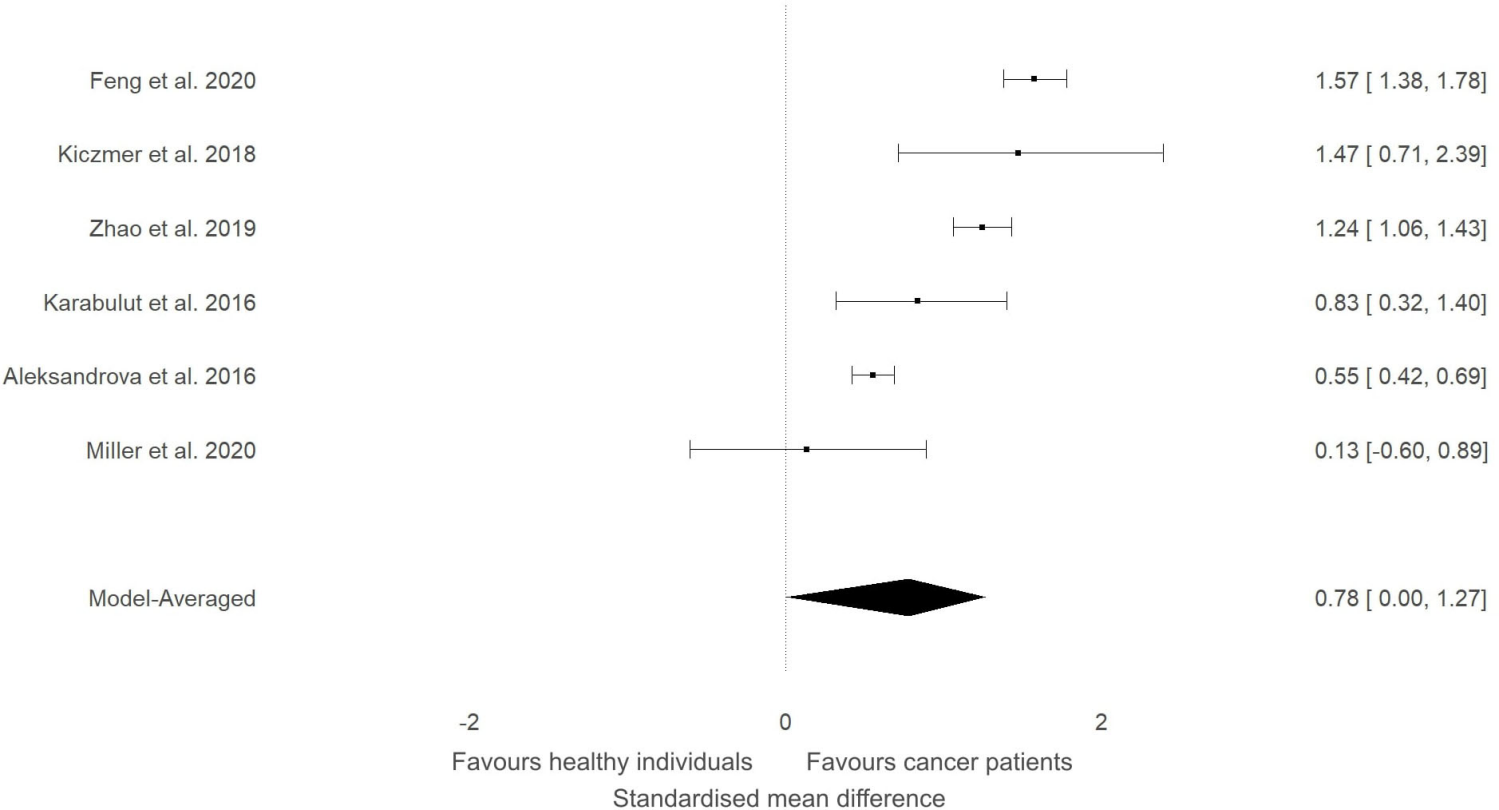
Forest plot showing meta-analytic results for studies that compared ITLN1 levels in people with gastrointestinal cancer and healthy individuals. Data are presented as SMD and 95% HDI.

**Figure 6:**
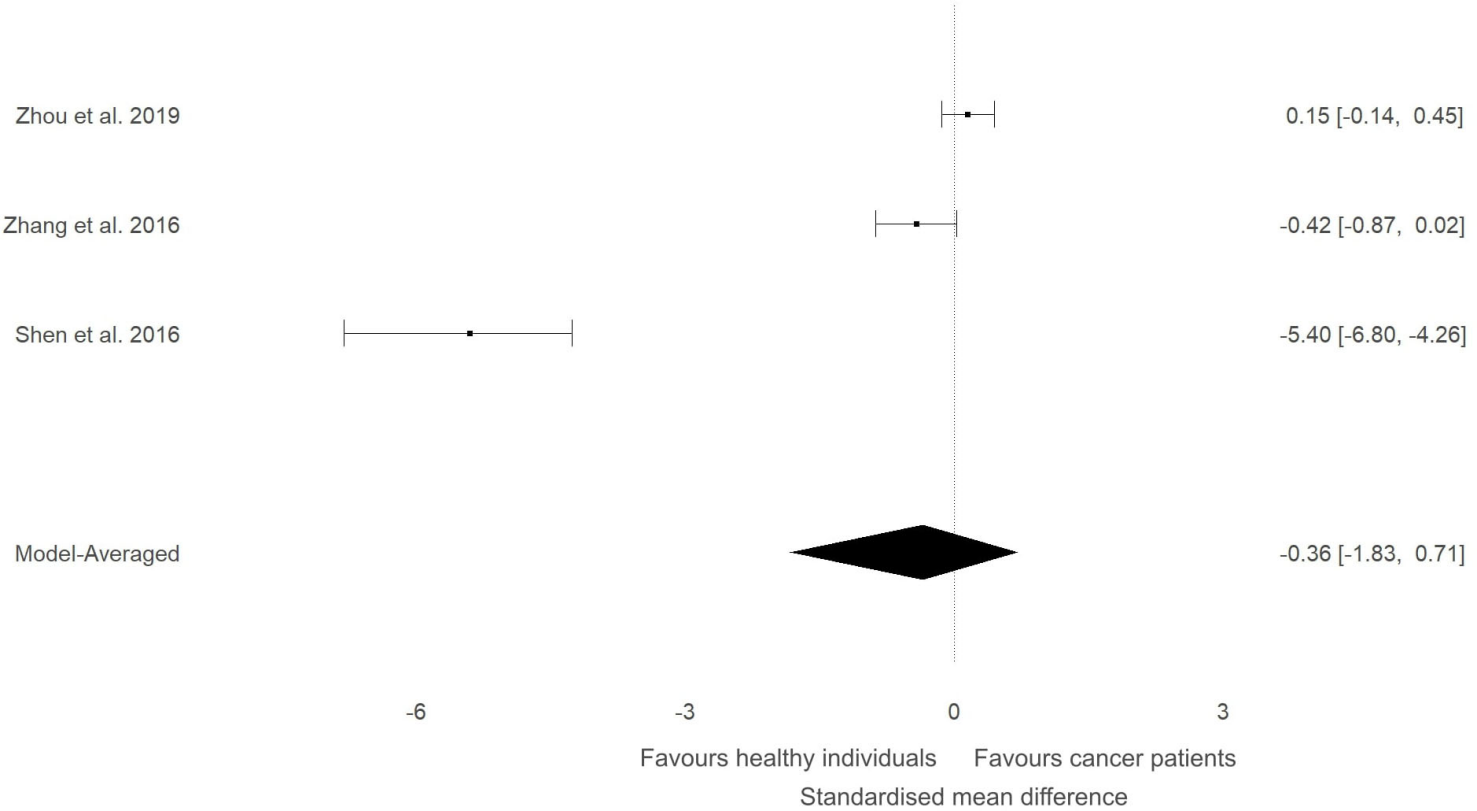
Forest plot showing meta-analytic results for studies that compared ITLN1 levels in people with urological cancer and healthy individuals. Model-averaged data are presented as SMD and 95% HDI.

**Figure 7:**
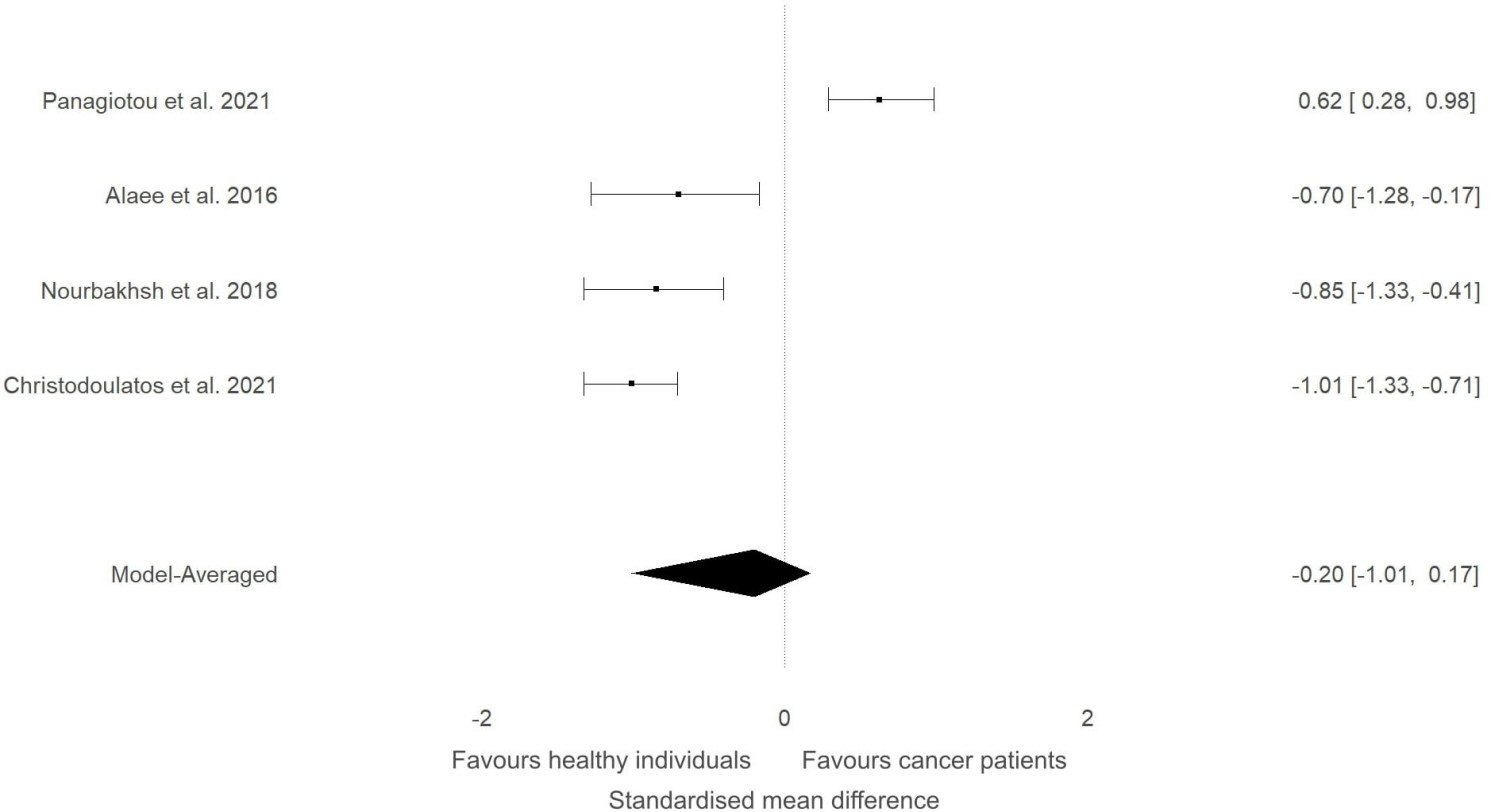
Forest plot showing meta-analytic results for studies that compared ITLN1 levels in people with breast cancer and healthy individuals. Model-averaged data are presented as SMD and 95% HDI.

In our previous narrative review (12) we described how the level of ITLN1 varies depending on cancer type. Consequently, a subgroup analysis was conducted to evaluate circulating ITLN1 differences between healthy participants and people with various types of cancer (i.e., gastrointestinal, urological and breast). As observed in **Figure 5**, there was strong evidence (BF=10.5) in favour of higher levels of ITLN1 in people with gastrointestinal cancer (i.e., colorectal, pancreatic, gastric) compared to healthy controls (SMD=0.77, 95%HDI: 0.00 to 1.27). However, there was only weak evidence that the concentration of ITLN1 was different between groups when individuals with urological cancer (**Figure 6**) were compared to healthy participants (SMD=-0.36, 95%HDI: -1.83 to 0.71, BF=1.2). Furthermore, women with breast cancer (**Figure 7**) had lower ITLN1 levels than healthy individuals (SMD=-0.20, 95%HDI:-1.01 to 0.17), but the available evidence was again weak (BF=0.9). All subgroup analyses showed very strong evidence in favour of high heterogeneity levels (BF>10000). The evidence for publication bias was weak for patients with gastrointestinal (BF=0.8), urological (BF=0.5) and breast cancer (BF=1.0).

### 3.3 Determining the level of ITLN1 in healthy individuals

In addition to the previously mentioned 15 studies that compared people with cancer to healthy individuals, another 148 studies measured ITLN1 levels in healthy individuals. The age of the healthy individuals was 44 ± 12. Moreover, 48 studies examined only females, 19 studies measured male participants only, while the remaining 96 studies evaluated a mix of females and males. The vast majority of the available literature as well as the results observed by the authors of the present report in clinical samples indicate that ITLN1 values are in the ng/ml range. Consequently, ITLN1 levels smaller than 1 ng/ml (i.e., difficult to measure) or greater than 2000 ng/ml (i.e., likely hyper-physiological) were excluded from the current analysis. After removing the studies containing unreasonable ITLN1 values, a total of 136 studies were subsequently examined. Of these, 52 were considered to provide high-quality data according to the criteria imposed by the quality assessment.

**Figure 8:**
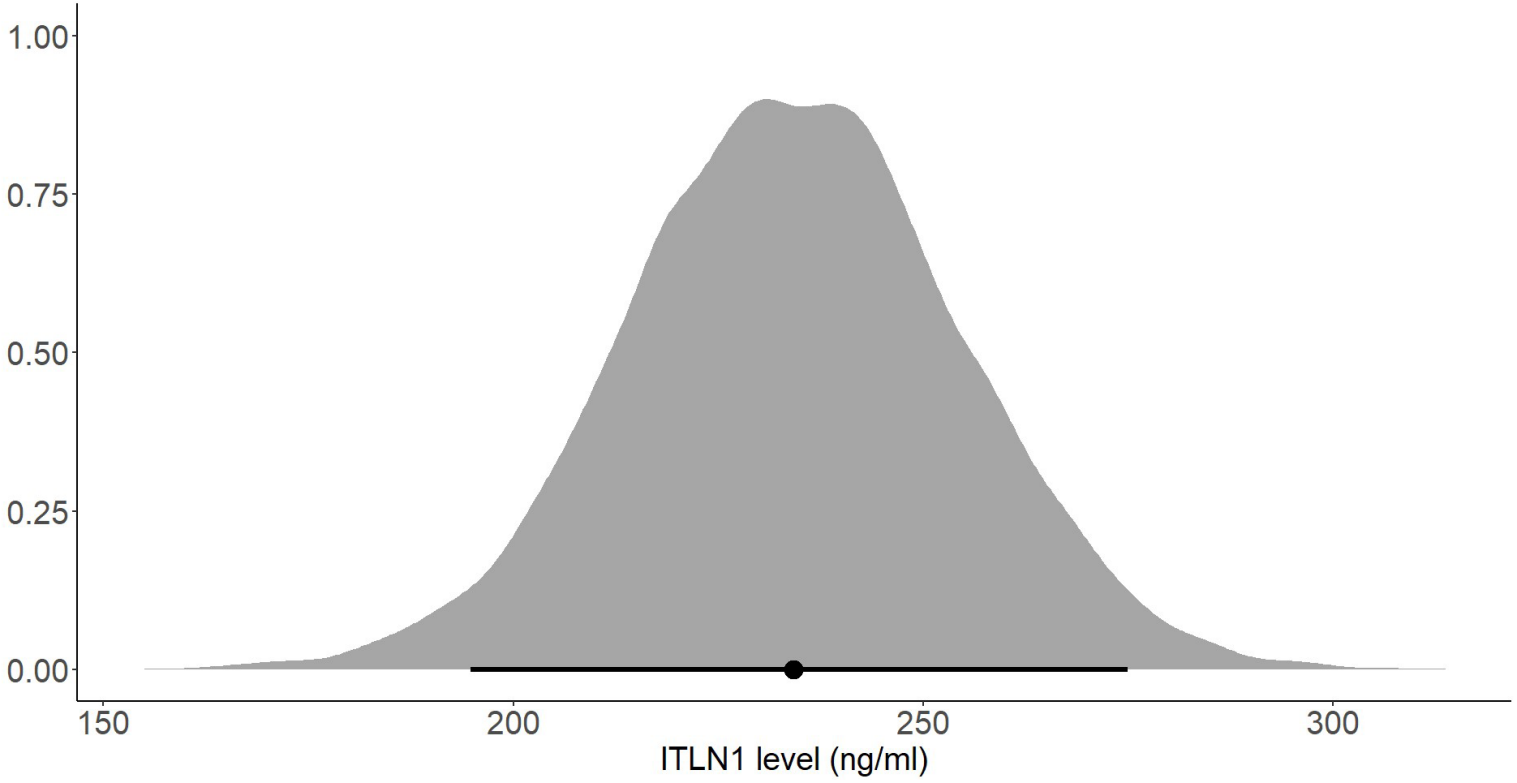
Posterior distribution of ITLN1 levels in healthy individuals determined from all studies included in the review. The point represents the mean of the posterior distribution and the line represents the 95% HDI.

**Figure 9:**
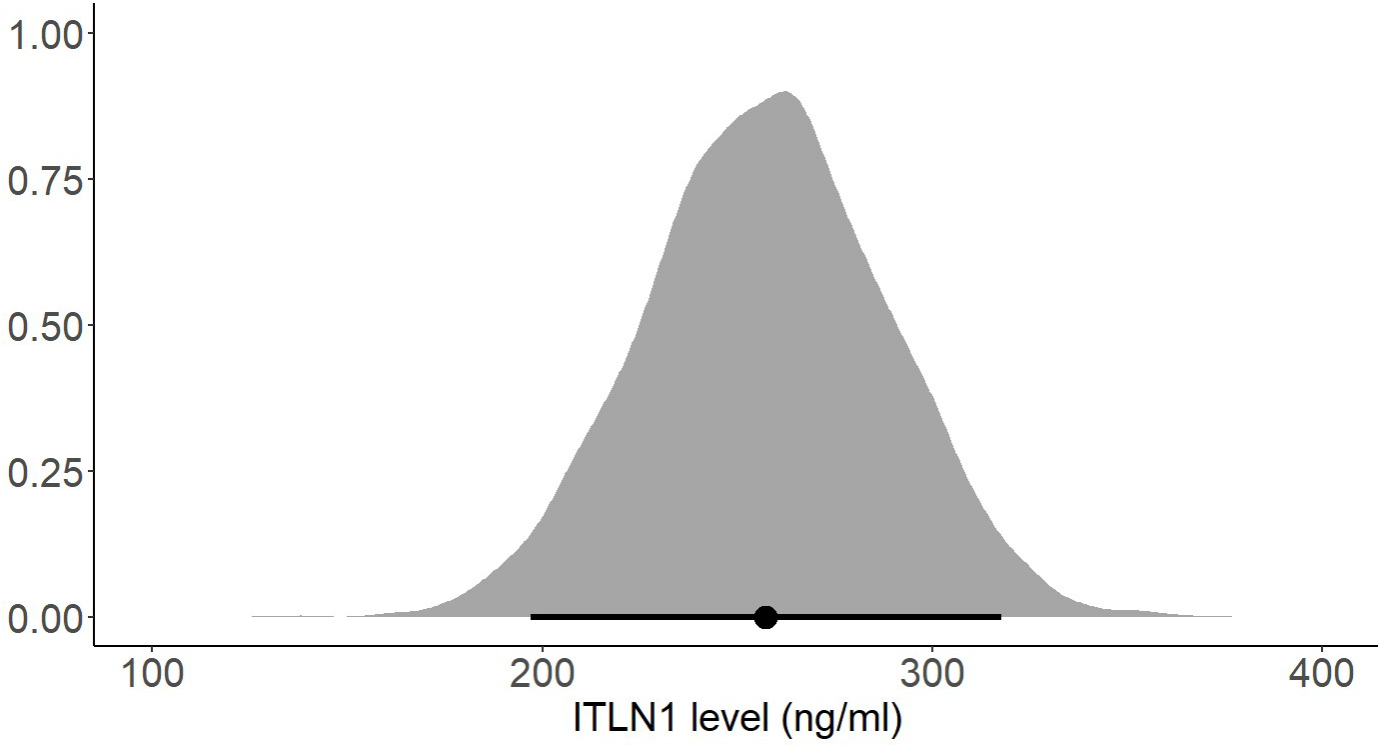
Posterior distribution of ITLN1 levels in healthy individuals determined from the high-quality studies included in the review. The point represents the mean of the posterior distribution and the line represents the 95% HDI.

The mean ITLN1 level in 10118 healthy individuals (**Figure 8**) was 234 ± 21 ng/ml (95%HDI: 193 to 275). The average concentration of ITLN1 was similar when examining high-quality studies (n=3301) only (**Figure 9**): 257 ± 31 ng/ml (95%HDI: 195 to 318). Based on the available data, it can be argued that circulating ITLN1 ranges from 195 ng/ml to approximately 318 ng/ml in healthy individuals.

**Figure 10:**
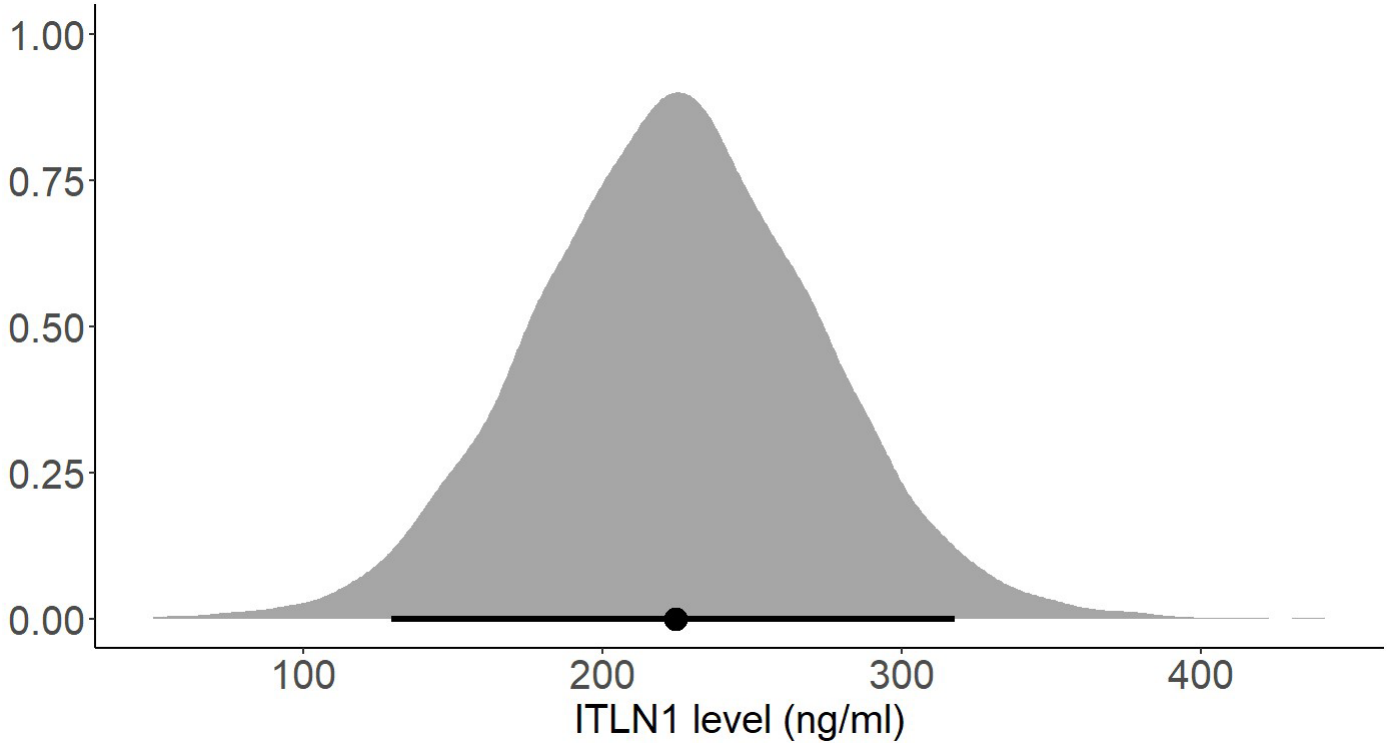
Posterior distribution of ITLN1 levels in healthy individuals with a BMI ≤ 25. The point represents the mean of the posterior distribution and the thick line represents the 95% HDI.

**Figure 11:**
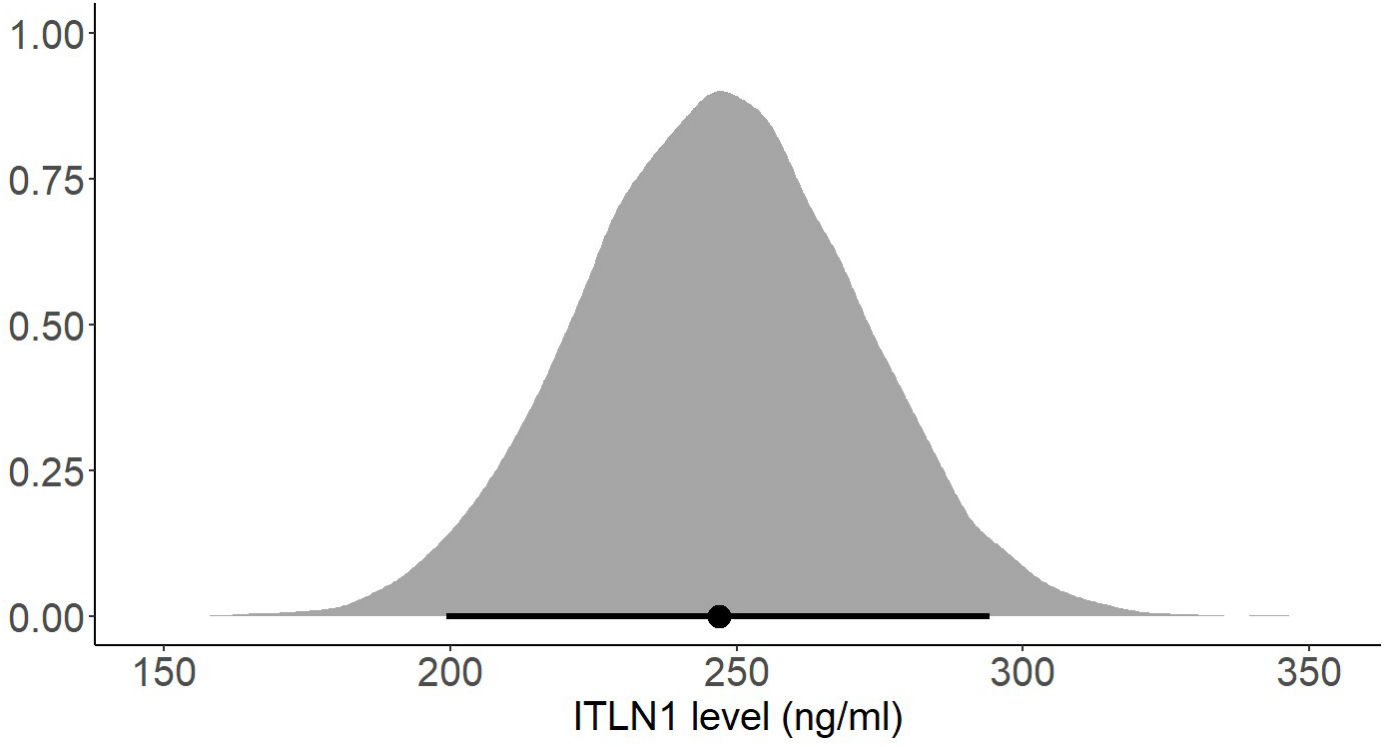
Posterior distribution of ITLN1 levels in healthy individuals with a BMI>25. The point represents the mean of the posterior distribution and the thick line represents the 95% HDI.

Several subgroup analyses were conducted to determine whether the participants’ gender or BMI affects ITLN1 concentrations. There was no substantial difference in ITLN1 levels between females (249 ± 34 ng/ml) and males (251 ± 53 ng/ml). Additionally, participants were grouped according to their BMI. Since none of the participants had a BMI<20, healthy individuals were divided into two groups: BMI ≤ 25 (**Figure 10**) and BMI>25 (**Figure 11**). Interestingly, ITLN1 concentrations were slightly lower in participants with BMI ≤ 25 (224 ± 48 ng/ml) than in participants with BMI>25 (246 ± 24 ng/ml).

## 4 Discussion

### 4.1 Main findings

The present study aimed to evaluate the differences in ITLN1 levels between people with cancer and healthy individuals and to establish the physiological concentration of circulating ITLN1 in healthy individuals. The meta-analysis suggested that circulating ITLN1 concentrations were not different between groups when multiple types of cancer were combined in the analysis (**Figure 2**). The same was observed in the sensitivity analysis (**Figure 3**) as well as in the analysis based on study quality (**Figure 4**). Another systematic review (20) also reported that circulating ITLN1 levels did not differ between groups when all types of cancer were included in the same analysis. Additionally, previous literature that examined the role of ITLN1 (7) reported that distinct types of cancer can differentially influence ITLN1 concentration. To examine this claim, a subgroup analysis was conducted by dividing the included studies based on cancer type. The analysis highlighted that people with gastrointestinal cancer had substantially higher levels of ITLN1 compared to healthy controls (**Figure 5**). Indeed, all studies that examined gastrointestinal cancers (n=6) observed that the healthy controls showed lower ITLN1 concentration and thus, the meta-analytical model suggested the presence of strong evidence in favour of this conclusion. Furthermore, there was weak evidence in favour of elevated ITLN1 levels when healthy individuals were compared to people with urological (**Figure 6**) or breast cancer (**Figure 7**). This confirms the observations we made in our previous narrative review (12) and indicates that the trajectory of circulating ITLN1 is influenced by the cancer type. Other research also emphasised that certain adipokines such as adiponectin (47), resistin (48) or leptin (49) were associated with obesity-related cancers. Therefore, it can be argued that ITLN1 behaves similarly since its levels are increased in patients with gastrointestinal cancer compared to healthy counterparts. An alternative explanation could be that gastrointestinal cancers are found in local proximity to the VAT depots and consequently, the tumour proximity might influence circulating ITLN1 levels by altering levels of locally produced ITLN1. This relationship, as well as the concentration of ITLN1 in urological and breast cancer should be examined by future research since only a limited number of studies have been published to date.

The current systematic review aimed to examine both circulating and tissue concentrations of ITLN1. Due to the low availability of data on tissue levels, only circulating concentrations were meta-analysed. Interesting findings were also reported by studies that measured ITLN1 in different tissues. Recently, our group analysed a cohort of people with upper gastrointestinal cancer (13) and reported that ITLN1 mRNA levels were higher in the VAT of people with cancer compared to healthy controls, but no difference was observed when SAT concentrations were compared between groups. Moreover, this study suggested that ITLN1 was a characteristic of cancer-associated weight loss. Other research (14) noted that ITLN1 protein expression was elevated in gastric cancer tissue compared to the normal gastric mucosa. Interestingly, some studies (50, 51) indicated that colorectal cancer patients with higher ITLN1 tumour concentrations had a better prognosis than those with lower levels. Therefore, the increased expression of circulating ITLN1 in gastrointestinal cancer patients compared to the lower levels observed in healthy individuals could be determined by the tumour and/or by the weight-loss specific to these types of cancer. The idea that higher ITLN1 tumour concentrations could have a protective role within cancer cohorts is also intriguing and further research should evaluate this hypothesis. Tissue concentrations of ITLN1 were also analysed in lung (52), ovarian (53) or breast cancer (10), but the available evidence is not sufficient for a comprehensive discussion about the adipokine’s effects and roles in these cancer types.

Using a Bayesian approach, the present meta-analysis also estimated HDIs for the mean level of circulating ITLN1 in healthy individuals (**Figures 8**, **9**). The mean concentration of ITLN1 was 243 ± 21 ng/ml when all studies were included in the analysis and 257 ± 31 ng/ml when considering high-quality studies only. Surprisingly, it was observed that ITLN1 was considerably variable even in the subgroup of studies that were of high quality (i.e., from 2 to 780 ng/ml). Thus, it can be argued that the adipokine’s variable levels were not caused by differences in the blood collection method or by dissimilarities in the assays used to quantify its concentration. A previous review that evaluated the biology and the role of ITLN1 in various diseases (7) also reported a wide range of values for circulating ITLN1 in healthy individuals (i.e., from 2 to 850 ng/ml). However, to date, no study has investigated potential reasons for the observed high degree of variability in circulating ITLN1. The findings of the current review could represent a starting point for future *in-vitro* and *in-vivo* work to better understand ITLN1 behaviour and function. Moreover, the levels of ITLN1 were not different between healthy females and males. Overweight and obese individuals (**Figure 11**) expressed marginally higher ITLN1 concentrations (246 ± 24 ng/ml) than individuals with a BMI ≤ 25 (224 + 48 ng/ml; **Figure 10**). Since VAT is the primary source of ITLN1, the elevated levels observed in individuals with BMI>25 could be attributed to excess overall adiposity. This contradicts prior research emphasising that circulating ITLN1 was lower in obese individuals (46, 54). Yet, the systematic review of Arab and colleagues (54) showed a high level of heterogeneity and evidence of publication bias in reports of the relationship between ITLN1 and overweight/obesity and, similar to the present study, also failed to observe any differences between groups when high-quality studies were evaluated. Additionally, despite the high number of participants included in the present review, the differences indicated by the meta-analytical model are minimal given the variable ITLN1 levels observed in healthy individuals. Also, since distinct systematic reviews observed different directions of the relationship between BMI and ITLN1 and given the small differences between groups, it could be argued the evidence is mixed and there is no substantive relationship between ITLN1 and BMI. Plausibly, there may be other factors influencing the relationships and future work should focus on discovering and understanding these mediators. We previously suggested that metabolic status may be the driver of ITLN1 levels rather than overweight/obesity per se (12). However, we did not have sufficient data to examine this hypothesis in the current study.

**Figure 12:**
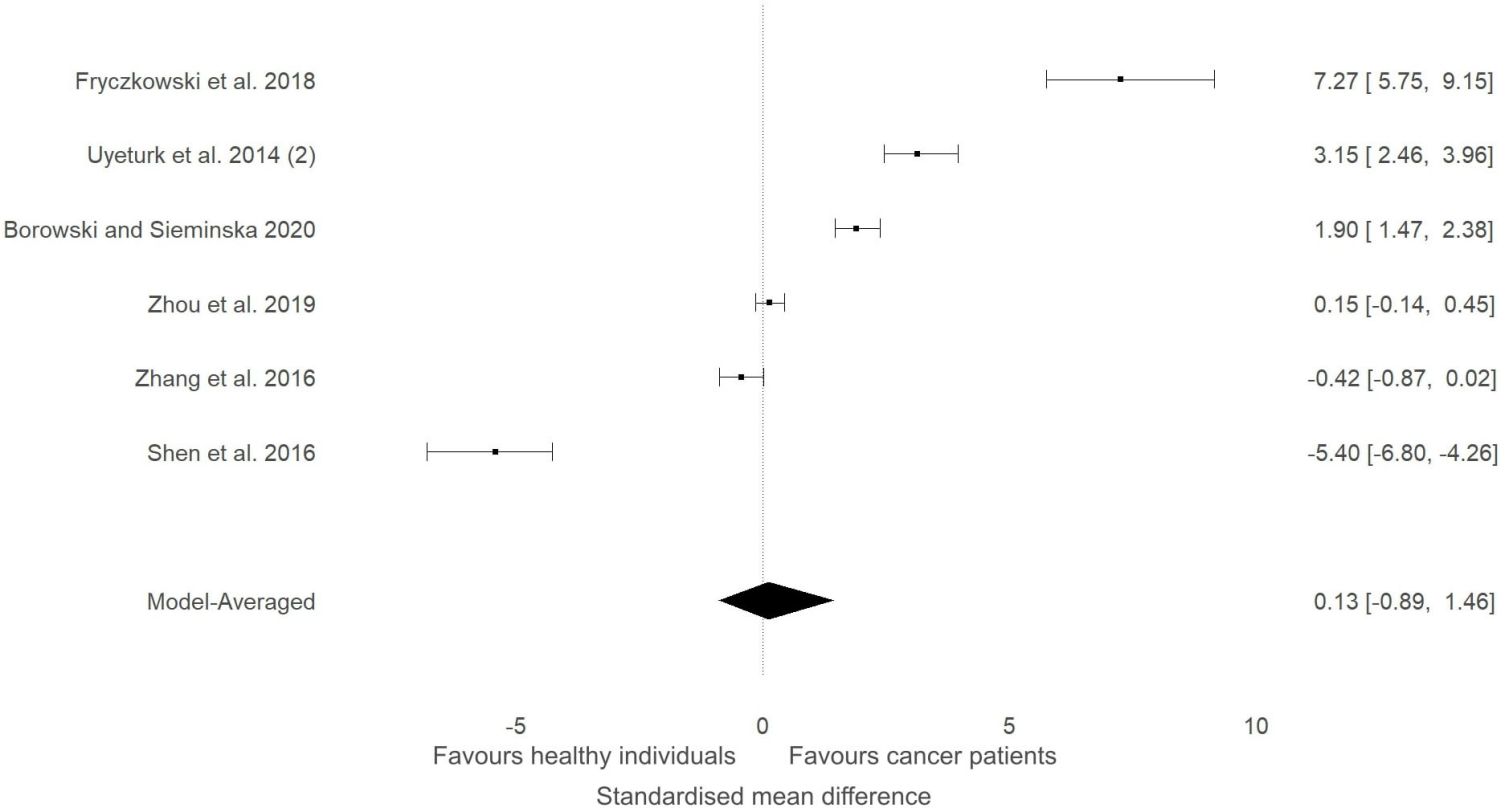
Forest plot showing meta-analytic results of the robustness check for the studies that compared ITLN1 levels in people with urological cancer and healthy individuals Three studies (Uyeturk et al. (50); Frczkowski et al. (51); Borowski and Seminska et al. (52)) did not include a control group and the mean ITLN1 of healthy individuals (234 ± 21 ng/ml) was used as a reference point in these instances. Model-averaged data are presented as SMD and 95% HDI.

Five studies that measured ITLN1 in cancer did not include a healthy control group and were consequently excluded from the meta-analysis (**Table 1**). The levels of circulating ITLN1 in the studies that examined people with prostate cancer (42, 44, 45) were overall higher than the mean ITLN1 concentration of healthy individuals (i.e., 257 ± 31 ng/ml). The studies assessing urological cancer that were included in the meta-analytic model (**Figure 6**) showed divergent results. It would be interesting to observe the extent to which the results of the subgroup analysis change if the previously mentioned studies (i.e., that did not include a control group) would be added to the statistical model. Consequently, the meta-analysis was updated and these studies (42, 44, 45) were added to the model (**Figure 12**). Since these studies failed to include a control group, the mean ITLN1 level of healthy individuals that was determined earlier (i.e., 234 ± 21 ng/ml) was used as a reference point. Following this robustness check (**Figure 12**), the overall ITLN1 difference between patients with urological cancer and healthy individuals was still low and not considerably different from the initial model (**Figure 6**). Similarly, one study (10) analysed patients with breast cancer without including a control group and reported values that were lower compared to the mean ITLN1 level of healthy individuals that was established earlier in this review. This observation is in accordance with the meta-analytical model that evaluated patients with breast cancer as three out of four of the included reported higher ITLN1 concentration in the control group. Furthermore, Tahmasebpour and colleagues (10) also measured gene expression and noticed that ITLN1 was downregulated in breast cancer tissue as opposed to adjacent normal tissue. To conclude, the majority of the studies examining circulating and tissue levels of ITLN1 in breast cancer patients indicate a tendency for higher concentration in healthy individuals and healthy tissues. However, the statistical model suggested that the available evidence is weak, and it is recommended that future studies examining ITLN1 concentration in cancer include suitable controls to allow effective comparisons in meta-analyses.

### 4.2 Limitations and methodological weaknesses

All meta-analytic models from the present review showed strong evidence in favour of heterogeneity and weak evidence of publication bias. The high degree of heterogeneity could be caused by the wide spread of ITLN1 values (from 1 to 750 ng/ml) or by the methodological variations observed across the literature. Indeed, there were multiple methodological issues observed in the studies included in this review and this is a major limitation of the available literature. Overall, only 33% of the studies in the meta-analysis comparing people with cancer to healthy individuals (**Figure 1**) and 38% of the studies included in the statistical model estimating the mean ITLN1 level (**Figure 9**) were of high quality. For the purpose of the present review, a high-quality study collected blood in the morning after an overnight fast, described the assay used to quantify ITLN1 and reported values that were in accordance with the characteristics of the assay (e.g., within detection limits). The studies that were not included in the high-quality subgroup failed to meet one, two or all of these standards. It can be argued that the criteria that must be met for inclusion in the high-quality subgroup (i.e., blood collection method, accurate description of the assay and precise methods of reporting results) should be standard practice in modern-day research and thus, the number of low-quality studies was exceptionally high. Similar methodological weaknesses were observed in a recent review conducted by our research group that looked at the relationship between cytokines and cancer cachexia (55). Poor data reporting and suboptimal descriptions of the methodology are too often encountered in the literature that evaluates the role of (adipo)cytokines in cancer (cachexia). Authors, reviewers and editors should aim to encourage transparency and promote basic methodological norms that facilitate the interpretation of the results and lead to more reliable conclusions in meta-analyses.

The current systematic review had several limitations. Firstly, only a few studies compared ITLN1 between healthy individuals and people with cancer. The subgroup assessments based on cancer type also included a limited number of studies and the data generated by these meta-analytic models should be interpreted with caution. Secondly, there is not enough research that evaluated the expression of ITLN1 in various tissues. This is particularly relevant in patients with cancer since the characteristics of different tissues could facilitate the understanding of a tumour’s malignant behaviour. Lastly, the screening process was conducted independently by one author. Although having only one reviewer is not recommended in systematic reviews, this study’s inclusion criteria were not strict, and the screening was conducted with a conservative approach. Since all studies that measured ITLN1 levels were included in the review, having a second reviewer would increase validity but would not improve the effectiveness of the screening.

Overall, it is challenging to conclude if circulating cytokine levels are clinically relevant in cancer or other medical conditions. The circulating ITLN1 concentrations we determined (13) in samples coming from healthy individuals (i.e., 2.3 ng/ml) and cancer patients (i.e., 2.8 ng/ml), using an ELISA kit (Amsbio, EH0564; notably no longer available), are substantially different from the average ITLN1 values discussed in this study. The range of circulating ITLN1 concentrations we recently observed in a sample of patients from the REVOLUTION trial (56) was also distinct from our previous observations (13) and from what was discussed earlier in the present study. Specifically, circulating ITLN1 was measured in patients with various types of cancer using a different ELISA kit (Abcam, ab269545) and the values ranged from 7 ng/ml to 48 ng/ml, with a mean level of 18 ng/ml. The high degree of variability observed in circulating (adipo)cytokine levels goes beyond ITLN1 as we highlighted in a recent systematic review (55). The concentrations of multiple cytokines (i.e., IL-6, TNF-α) were heterogenous in both healthy individuals and cancer patients. Furthermore, previous research indicated that several cytokines (e.g., IL-1β) are often undetectable (57, 58) and this was also observed in our laboratory when we analysed patients from the REVOLUTION trial. Interestingly, multiple studies analyse the detectable levels without discussing the possible reasons for values being below the limit of detection for some samples. The rate of false positives in meta-analyses and systematic reviews will drastically increase if these practices are repeatedly used across the literature. Another aspect of particular interest is the mechanism of action of ITLN1. Our recent narrative review (12) suggested that ITLN1 can activate the PI3K/Akt pathway and discussed other potential mechanisms responsible for its effects. As we previously highlighted (12), the available evidence is inconclusive and no further relevant mechanistic studies have been published in the meantime. This research gap should be thoroughly explored for both ITLN1 and other relevant (adipo)cytokines (e.g., IL-1β) with cell culture experiments and animal models. To conclude, the clinical relevance of circulating (adipo)cytokine levels remains somewhat uncertain and future studies should explore the mechanisms that determine the high degree of variability and whether tissue concentrations (e.g., tumour) are a more relevant measurement.

### 4.3 Conclusions

Circulating ITLN1 did not show any difference between groups when combined tumour types were considered in the same analysis. Yet, the concentration of the adipokine was considerably higher in patients with gastrointestinal cancer compared to healthy individuals, making it a potential therapeutic target. ITLN1 concentrations may be overall lower in breast cancer patients compared to controls, but the data were not sufficient to draw a strong conclusion. Another key finding of the present systematic review was the estimated mean ITLN1 level in healthy individuals: 257 ± 31 ng/ml. This value could be a useful starting point for studies that aim to examine the role and the behaviour of ITLN1 using *in-vitro* and *in-vivo* models. Future research on circulating ITLN1 and other biomarkers should improve methodological quality by adhering to basic norms. The blood collection method, the assay used to quantify ITLN1 and the complete set of results should be reported and thoroughly described. Efforts should be made to monitor the evolution of ITLN1 during cancer by implementing longitudinal study designs.

## Data availability statement

The original contributions presented in the study are included in the article/**Supplementary Material**. Further inquiries can be directed to the corresponding author.

## Author contributions

DP: conceptualisation, methodology, data analysis, writing the drafts, and the final version of the manuscript, editing. TD: conceptualisation, writing the drafts and the final version of the manuscript. RS: conceptualisation, writing the drafts and the final version of the manuscript. IG: conceptualisation, methodology, data analysis, writing the drafts and the final version of the manuscript, editing, supervision.
