# Supplementary material for: A systematic review and Bayesian meta-analysis assessing intelectin-1 in cancer patients and healthy individuals
Source: Front Oncol. 2024 Feb 28;14:1198555. doi: 10.3389/fonc.2024.1198555 (PMC10933003; doi:10.3389/fonc.2024.1198555)
Supplement: Supplementary file 1 [file DataSheet_1.docx]

**Supplementary materials**

**Search strategy**

*Ovid MEDLINE– 1946 to date*: 759 results

intelectin* {including related terms} or omentin* {including related terms}

*Ovid EMBASE – 1946 to date*: 1290 results

Intelectin*.mp or omentin*.mp

*Web of Science – Core collection*: 948 results

TS=intelectin* or TS=omentin*

*CINAHL – on EBSCO host*: 142 results

intelectin* or omentin*

*CENTRAL – Cochrane Library*: 80 results

(intelectin*):ti,ab,kw and (omentin*):ti,ab,kw

**R code and datasets**

The datasets, the R script used to analyse the data and the data collection form can be found in the following GitHub repository: <https://github.com/rpaval/bayesian_meta_analysis>
